# Supplementation with ready-to-use therapeutic food has no effect on adverse outcomes among undernourished children aged 6–59 months with severe pneumonia

**DOI:** 10.3389/fnut.2025.1507360

**Published:** 2025-06-06

**Authors:** Damalie Nalwanga, Elisa Giallongo, Victor Musiime, Sarah Kiguli, Peter Olupot Olupot, Florence Alaroker, Robert Opoka, Abner Tagoola, Mainga Hamaluba, Christabel Mogaka, Eva Nabawanuka, Charles Karamagi, André Briend, Kathryn Maitland

**Affiliations:** ^1^Department of Paediatrics and Child Health, School of Medicine, College of Health Sciences, Makerere University, Kampala, Uganda; ^2^Paediatrics Research Group, Makerere University Lung Institute, Kampala, Uganda; ^3^Intensive Care National Audit & Research Centre, London, United Kingdom; ^4^Joint Clinical Research Centre, Kampala, Uganda; ^5^Mbale Clinical Research Institute, Mbale, Uganda; ^6^Soroti Regional Referral Hospital, Soroti, Uganda; ^7^Aga Khan University, Medical College East Africa, Nairobi, Kenya; ^8^Jinja Regional Referral Hospital, Jinja, Uganda; ^9^KEMRI Wellcome Trust Research Programme, Kilifi, Kenya; ^10^Clinical Epidemiology Unit, Department of Internal Medicine, School of Medicine, College of Health Sciences, Makerere University, Kampala, Uganda; ^11^Tampere Center for Child, Adolescent and Maternal Health Research, Faculty of Medicine and Health Technology, Tampere University and Tampere University Hospital, Tampere, Finland; ^12^Department of Nutrition, Exercise and Sports, Faculty of Science, University of Copenhagen, Frederiksberg, Denmark; ^13^Department of Infectious Disease, Institute of Global Health and Innovation, Imperial College London, London, United Kingdom

**Keywords:** severe pneumonia, undernutrition, nutritional intervention, African children, adverse outcomes

## Abstract

**Objectives:**

To investigate the effect of supplementation with ready-to-use therapeutic food (RUTF) on adverse outcomes among undernourished children aged 6–59 months with severe pneumonia.

**Methods:**

This secondary analysis of the COAST-Nutrition (ISRCTN10829073) included children hospitalized for severe pneumonia in Uganda and Kenya. Undernutrition was defined as having either a weight-for-age z score, height-for-age z score, or weight-for-height/length z score below the median of the WHO reference population (< 0) or mid-upper arm circumference (MUAC) below 13.5 cm. Participants were randomized to receive 1 sachet of RUTF daily for 8 weeks in addition to the usual diet (intervention) or usual diet alone (control). The primary composite outcome for adverse events was any one of mortality, re-admission, or deterioration of nutritional status by day 90 of follow-up.

**Results:**

Of 846 main trial participants, 741 (88%) met the inclusion criteria (intervention: 374 versus control: 367). Of 687 (93%) participants in whom the primary outcome was assessed, 370 (54%) experienced an adverse event, [intervention: 184/348 (53%) versus control: 186/339(54%)]. There was no difference in the primary outcome between groups, aOR 0.92 (95% CI 0.68, 1.24), *p* = 0.572. Adverse outcome risk reduced with increasing age, aOR 0.53, (95% CI 0.45, 0.62), *p* < 0.001.

**Conclusion:**

RUTF supplementation did not reduce the high frequency of adverse outcomes in children aged 6–59 months following hospital admission with severe pneumonia. Nutritional support directly targeting metabolic needs post-pneumonia should be considered in the future.

**Clinical trial registration:**

ISRCTN10829073, PACTR202106635355751.

## Background

Despite a significant reduction in the incidence of childhood pneumonia due to the roll-out of immunization, uptake of HIV prevention and treatment and some reduction in the major risk factors (malnutrition, non-exclusive breastfeeding, crowding, and indoor air pollution) pneumonia remains a major public health challenge, particularly in Africa (1–3). Moreover, the reduction of pneumonia mortality lags behind other common pediatric infectious diseases in lower to middle-income countries (LMICs) (2). In 2021, over 37 million pneumonia episodes were reported among children under 5 years, with an incidence rate of 5,750 per 100,000 (1). The majority of pneumonia cases occur in LMICs (1, 3), where undernutrition is also persistently prevalent (4). Undernutrition in the form of wasting, stunting, or being underweight is one of the top ten risk factors for the disease burden among children under 5 years (1). Undernutrition significantly increases the risk of developing severe pneumonia among children (5). Undernutrition also results in a higher frequency of adverse outcomes including mortality (6–9), deterioration in nutritional status (10–13), recurrent infections, and repeated hospital readmissions (14, 15) among children.

Pneumonia is the commonest infectious cause of mortality in children, accounting for over 14% of deaths among children under 5 years, and approximately 740,180 children in 2019 (3, 16). Children with pneumonia may die directly as a result of complications of the disease itself such as hypoxemia (17, 18), or indirectly via secondary infections related to immune compromise (19). Pneumonia also increases the risk of deterioration of children’s nutritional status via increased metabolic demands to support physiological functions such as hyperthermia and increased work of breathing at a time when the children have reduced appetite and are unable to feed adequately (20, 21). Deterioration in nutritional status further compromises their immunity with resultant increased risk of infections, re-admission to hospital, and potentially death (10, 11, 14, 15, 21).

The World Health Organization (WHO) under the Integrated Global Action Plan for the Prevention and Control of Pneumonia and Diarrhea (GAPPD) recommends “continued feeding” in the “diagnose and treat” element (22). However, the nutritional requirements in a child with pneumonia are likely to be higher than those of a well-child (10, 11), and if not addressed could increase the risk of deterioration in nutritional status following the pneumonia episode. Children recovering from pneumonia are likely to require more energy to recover from deficits resulting from increased metabolic demands and anorexia during the illness.

According to the joint consultation of the WHO, United Nations Children’s Fund, World Food Program, and the United Nations High Commissioner for Refugees in October 2008, the desirable nutrient intakes for recovery from MAM are probably in the range between the recommended nutrient intakes for well-nourished children and the intakes recommended in the recovery phase for those with severe malnutrition (23, 24). The options available in Africa to meet these energy requirements are ready-to-use supplementary food (RUSF) and Ready-to-Use Therapeutic Food (RUTF). While RUSF is only available for community nutritional programs, RUTF is used for the follow-on nutritional rehabilitation of children hospitalized with severe acute malnutrition. Previous studies suggest that the provision of one sachet per day (500 kcal) for children under 5 years with moderate malnutrition (defined as a mid-upper arm circumference (MUAC) 11.5 cm − < 12.5 cm) could provide over 49% of the daily energy requirements of children with MAM (25), and improve outcomes (26). Considering the actual nutrient requirement for children with severe pneumonia population is unknown, there is no macronutrient intervention uniquely designed for them. However, nutritional requirements during and after recovery from a pneumonia episode are likely to lie between the daily recommended allowance for age and the requirement for children with moderate malnutrition. RUTF could therefore be extended to children at high risk of undernutrition/malnutrition, such as children recovering from severe pneumonia.

RUTF is a peanut-based paste, specifically designed for children with malnutrition, which provides additional energy, protein, fat and micronutrients. RUTF has been used safely for the treatment of moderate acute malnutrition (MAM) with significantly higher patient recovery, reduced non-recovery, and better weight gain when compared to patients treated with blended foods (27, 28). When compared to nutritional counseling or micronutrient supplementation only, RUTF is associated with better anthropometric outcomes (29). A short course of RUTF has also been considered for African children with acute infections to improve growth with varying results in two cohorts (30, 31). The Children’s Oxygen Administration Strategies-Nutrition Trial (COAST-Nutrition) was designed to investigate whether supplementary feeding with ready-to-use therapeutic food (RUTF) could provide additional nutritional requirements and improve global outcomes in children hospitalized with severe pneumonia across the anthropometric spectrum (32). The trial showed that 90-day mortality and the increment in mid-upper-arm circumference (MUAC) were similar among the children who received RUTF compared to those who did not receive it.

We hypothesized that 1 sachet of RUTF given daily for 8 weeks in addition to the usual diet to undernourished children aged 6–59 months with severe pneumonia would reduce the risk of adverse outcomes (mortality, deterioration in nutritional status, and readmission). If shown to benefit children with undernutrition following admission with pneumonia, RUTF could potentially be rapidly deployed for nutritional support in children with pneumonia.

## Methods

### Study design and setting

The study was a secondary analysis of a phase II multicenter open-label randomized clinical trial (ISRCTN10829073), the Children’s Oxygen Administration Strategies-Nutrition Trial (COAST-Nutrition) study (32). It was conducted at five sites in Uganda: Mbale, Soroti, Jinja, and Masaka Regional Referral Hospitals, and Kenya: Kilifi County Hospital between August 2018 and April 2022 with ethical approval from Imperial College London Research Ethics Committee (15IC3100); School of Medicine Makerere University REC (2020–155) in Uganda; and KEMRI Scientific and Ethics Review Unit C 215/4109 in Kenya.

### Participants and materials

The main trial included children aged 6 months to 12 years admitted with WHO signs and symptoms of severe pneumonia (33) with hypoxemia (pulse oximetry SP0_2_ < 92%) whose parents consented to participate in the study, and excluded those with severe acute malnutrition (MUAC < 11.5 cm and/or edema), known chronic lung disease, or congenital cardiac disease.

In this analysis, we only included children aged 6–59 months with undernutrition. Undernutrition was defined as having either a weight-for-age z score, height-for-age z score, or weight-for-height/length z score below the median of the WHO reference population (< 0) or mid-upper arm circumference (MUAC) below 13.5 cm (34). We considered a higher cut-off for undernutrition due to the known risk of undernutrition in this population (10, 11), and the high prevalence of inadequate dietary diversity in LMICs based on the WHO standards (35, 36).

### Outcome measures

The primary outcome measure was a composite of any one of the 3 adverse outcomes associated with severe pneumonia: mortality, re-admission, and deterioration of nutritional status, during 90 days of follow-up. Deterioration of nutritional status was defined as a drop in the WHO anthropometric range from baseline such as (i) Drop of z score from 0 - ≥ −1 to either −1- ≥ −2, −2 - ≥ −3, or to <−3, or (ii) Drop in MUAC from 12.5–13.5 to either 11.5–13.5 or to < 11.5 cm (34). The methods for assessing weight, length/height, and MUAC have been described elsewhere (32). Secondary outcomes included the composite of adverse outcomes at 180 days of follow-up and the separate individual adverse outcomes at 90 and 180 days of follow-up.

### Participant recruitment and follow-up

Participants were screened for eligibility for the study at the emergency units of the respective facilities by a study nurse. Baseline clinical characteristics (history and examination) and laboratory investigations were assessed at the point of enrolment into the parent trial (0 h) and entered into a case report form. Participants were managed for severe pneumonia according to the WHO pneumonia treatment guidelines (33), and randomized at 48 h post-admission (and trial entry) to receive either the intervention, i.e., one 92 g sachet (500 Kcal) per day of lipid-based nutrient supplement with multivitamins (RUTF) for 56 days (8 weeks) in addition to their usual diet (intervention), or the usual diet alone (control).

The intervention was started as soon as children were well enough to tolerate oral feeds. Children in both the intervention (RUTF) and control arms received nutritional education using the standard WHO Integrated Management of Childhood Illness (IMCI) Information, Education and Communication (IEC) materials (37). Children were followed up at days 28, 90, and 180 during which time those in the intervention arm continued to receive RUTF supplies on their visit days until they completed 8 weeks. Assessment of nutritional status was conducted at each follow-up time point. Data on readmission and death was collected as and when the events occurred during the study period. Adherence to the intervention was done on subsequent visits using parent/guardian reports and a count of the remaining RUTF packets compared to the number supplied (32). Details of participant randomization, allocation concealment, management, and follow-up are laid out in the published main trial protocol (38). Participants and outcome evaluators were not blinded to the intervention.

### Sample size estimation

This analysis was done in a sample of participants from the parent trial who fulfilled the selection criteria. We estimated that a sample size of 752 (376 in each group) would give a confidence interval width of 0.02 for a confidence level of 0.95 and a difference of 58% in the proportion of events between the intervention and control group (39).

### Data analysis

Baseline characteristics were summarized using frequencies and percentages (%), or medians and interquartile ranges (IQR). Analysis was by Intention-to-Treat with the primary analysis population defined as all patients who were randomized and had a primary outcome recorded (complete case analysis). The primary composite outcome was compared between groups using a multilevel mixed-effect logistic regression with the site as a random factor. Results were presented as odds ratios, tested with a two-sided *p*-value of 0.05, and a 95% confidence interval. We conducted an exploratory analysis for the factors associated with experiencing the composite adverse outcome by the end of the study follow-up (day 180). The baseline characteristics of participants who experienced the composite adverse outcome by day 180 of follow-up were summarized using frequencies and percentages (%), or medians and interquartile ranges (IQR). Factors associated with the composite adverse outcome were analyzed using logistic regression and adjusted for age, sex, and pediatric emergency triage (PET) score (40). The PET score is a risk score that is composed of 8 variables including temperature, heart rate, capillary refill time, level of consciousness, severe pallor, respiratory distress, lung crepitations, and weak pulse volume. The risk score was developed using data from severely ill African children who participated in the Fluid as Expansive Supportive Therapy (FEAST) trial to identify children at high risk of mortality and performed well against other published risk scores (40). It ranges from 1 to 10 with increasing severity of illness. Analyses were conducted using STATA version 18.0.

## Results

**Figure 1:**
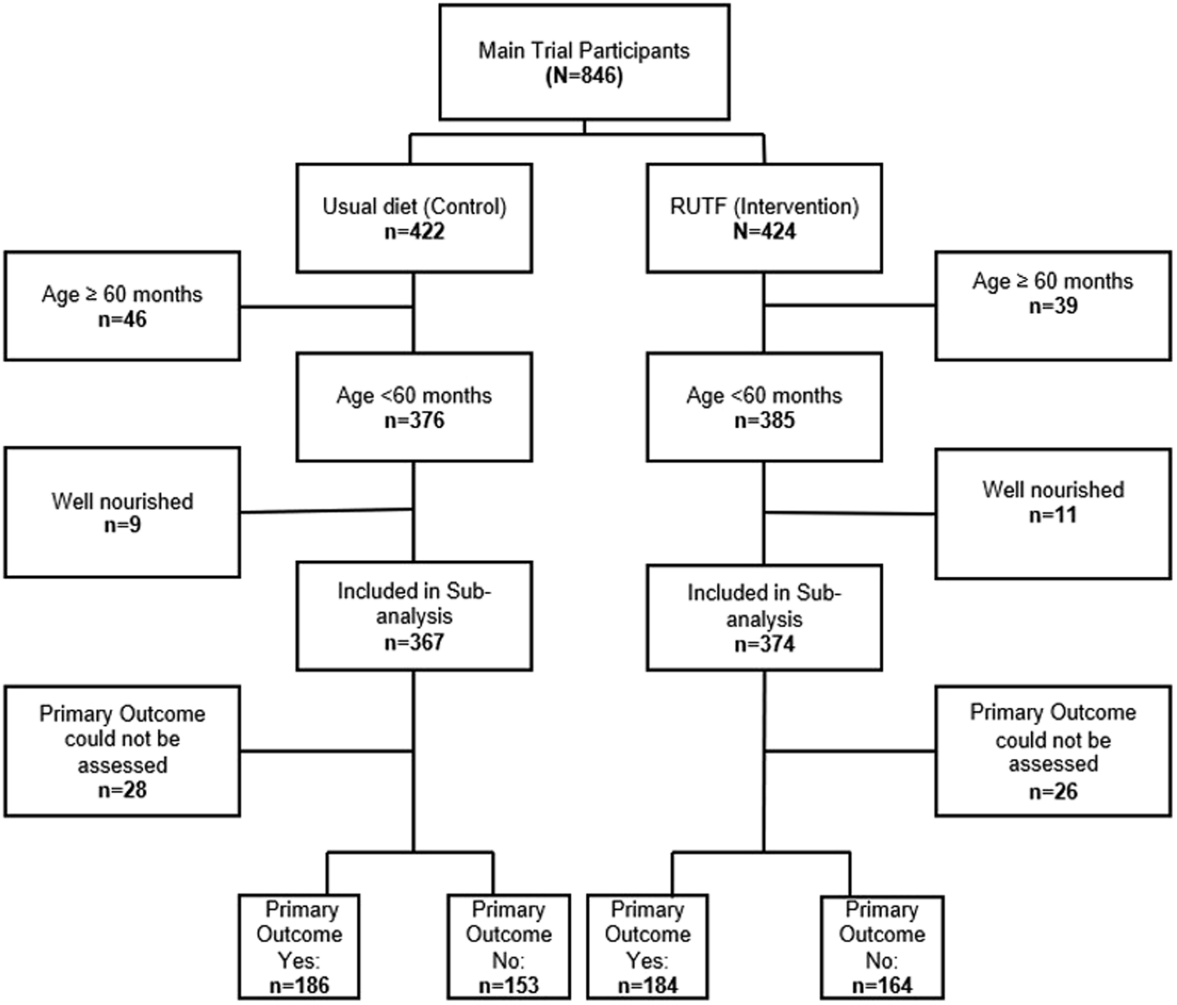
Participants selection flow diagram.

We included 741 undernourished participants aged 6–59 months; 374 in the intervention (RUTF) arm and 367 in the control (usual diet) arm (Figure 1). Overall, the median age was 16 (IQR 9.26) months and the majority of participants were male (n = 429, 57.9%). The median baseline weight-for-age z score was −1 (IQR – 2.0), height-for-age z score −1 (IQR – 2.0), and weight-for-height z score −0 (IQR – 1.0). The median MUAC was 14 cm (14, 16), and the majority (714, 96.4%) had MUAC ≥12.5 cm. The majority of the participants were afebrile (402, 54.3%), hypoxemic with median oxygen saturation of 89% (87.90), tachypneic (672, 90.7%), and alert (701, 94.6%). The majority of the participants’ caretakers reported difficulty in breathing at baseline (727, 98.1%), and that they had received oral antibiotics or injections before presentation to the hospital (399, 54.9%). On examination, the majority of the participants had chest indrawing (682, 92.2%), and crepitations (571, 77.1%), while 20% (*n* = 148) were grunting and 29.4% (*n* = 217) had an audible wheeze. A quarter of the participants (*n* = 178) had positive malaria rapid diagnostic tests, and 63 (8.6%) had positive malaria blood films. Eleven participants (1.6%) were HIV positive at admission and 74 (10.4%) were found to have sickle cell disease. The median PET severity score for included participants was 2 (IQR 1.3) with the majority of the participants having radiological evidence of pneumonia (430, 58.8%). Baseline characteristics were equally distributed between intervention and control arms (Table 1).

**Table 1:** Baseline characteristics (day of admission) for participants included in this secondary analysis, Uganda and Kenya 2018–2022.

Parameter; *n* (%) or Median (Interquartile range) as stated	Usual diet (*N* = 367)	RUTF (*N* = 374)	All patients (*N* = 741)
Median age (months)	16 (9, 27)	16 (9, 26)	16 (9, 26)
Male sex	218 (59.4)	211 (56.4)	429 (57.9)
Median weight-for-age baseline z score	−1 (−2, 0)	−1 (−1, 0)	−1 (−2, 0)
Median height-for-age baseline z score	−1 (−2, 0)	−1 (−2, 0)	−1 (−2, 0)
Median weight-for-length/height baseline z-score	−0 (−1, 0)	−0 (−1, 0)	−0 (−1, 0)
Median mid upper arm circumference (cm)	14 (14, 15)	15 (14, 16)	14 (14, 16)
Mid upper arm circumference ≥12.5 (%)	355 (96.7)	359 (96.0)	714 (96.4)
Fever (temperature >37.5 C)	165 (45.0)	174 (46.5)	339 (45.7)
Median initial Oxygen Saturation (SPO2)	89 (87, 90)	89 (87, 90)	89 (87, 90)
Median respiratory rate (breaths per minute)	56 (48, 62)	56 (49, 64)	56 (48, 64)
Age-adjusted tachypnea	326 (88.8)	346 (92.5)	672 (90.7)
Age-adjusted bradycardia	1 (0.3)	2 (0.5)	3 (0.4)
Age-adjusted severe tachycardia	93 (25.3)	86 (23.1)	179 (24.2)
Age-adjusted decompensated shock (severe hypotension)	1 (0.3)	0 (0.0)	1 (0.1)
Compensated shock (signs of impaired perfusion)	160 (43.6)	159 (42.5)	319 (43.0)
Responsiveness: Alert (%)	344 (93.7)	357 (95.5)	701 (94.6)
Signs of current febrile illness (temperature>37.5 or temperature <36)	172 (46.9)	182 (48.7)	354 (47.8)
History of fever	333 (90.7)	347 (92.8)	680 (91.8)
Difficulty breathing	362 (98.6)	365 (97.6)	727 (98.1)
Vomiting or diarrhea	117 (32.1)	118 (31.6)	235 (31.8)
Oral antibiotics or injections of antibiotics	204 (56.8)	195 (53.0)	399 (54.9)
Used Inhalers or oral steroids	16 (4.4)	16 (4.3)	32 (4.3)
In-drawing	340 (92.6)	342 (91.7)	682 (92.2)
Grunting	70 (19.1)	78 (20.9)	148 (20.0)
Crackles/crepitations on auscultation	281 (76.6)	290 (77.5)	571 (77.1)
Audible wheeze on auscultation	112 (30.5)	105 (28.2)	217 (29.4)
Signs of dehydration (sunken eyes or decreased skin turgor)	9 (2.5)	8 (2.2)	17 (2.3)
Gestation at birth ≥37 weeks (%)	346 (95.3)	353 (95.1)	699 (95.2)
Severe anemia	20 (5.5)	21 (5.7)	41 (5.6)
Leukocytosis	206 (56.4)	215 (58.3)	421 (57.4)
Lactate ≥5 mmol/L	9 (2.5)	11 (3.0)	20 (2.8)
Positive malaria rapid diagnostic test	277 (75.9)	279 (75.6)	556 (75.7)
Positive malaria blood film	37 (10.1)	26 (7.0)	63 (8.6)
Positive for HIV at admission	5 (1.4)	6 (1.6)	11 (1.5)
Sickle cell disease	34 (9.7)	40 (11.1)	74 (10.4)
Radiology evidence of pneumonia	206 (56.7)	224 (60.9)	430 (58.8)
Conscious level: Pain or voice	19 (82.6)	16 (94.1)	35 (87.5)
Breastfed	332 (90.7)	347 (92.8)	679 (91.8)
Immunization up-to-date	232 (89.2)	242 (90.6)	474 (89.9)
Moderate malnutrition	12 (3.3)	15 (4.0)	27 (3.6)
Severe hypoxemia (Oxygen saturation less than 80%)	21 (5.7)	26 (7.0)	47 (6.3)
Cardiac disease, congenital or other (%)	0 (0.0)	2 (0.5)	2 (0.3)
Median paediatric emergency triage (PET) score	2 (1, 3)	2 (1, 2)	2 (1, 3)

Overall, the primary outcome could not be assessed among 54 (7.2%) participants (Figure 1). The total number of participants who experienced the primary outcome was 370 (49.9%). There was no difference in the occurrence of the primary outcome between the intervention (184/348, 52.9%) and the control arms (186/339, 54.9%) with an adjusted odds ratio of 0.92 (95% CI 0.68, 1.24), *p* = 0.572 (Table 2). Adverse outcomes experienced by day 90 were deterioration in nutritional status (48%, *n* = 318), mortality (3%, *n* = 23) and readmission to hospital (8%, *n* = 56). There was no difference between the intervention and control groups when the separate components of the primary outcome at day 90 of follow-up were considered. By day 180 of follow-up, 449 (69.5%) participants had experienced the composite of adverse outcomes: 225/334 (67.4%) in the intervention and 224/312 (71.8%) in the control arm. There was no significant difference in the occurrence of the composite outcome between the intervention and control arms [adjusted odds ratio 0.80 (95% CI 0.57, 1.13)], or in the separate components by day 180 of follow-up (Table 2).

**Table 2:** Primary and secondary outcomes of undernourished children with severe pneumonia by randomization arm, Uganda and Kenya 2018–2022.

Parameter, *n*/*N* (%)	Usual diet	RUTF	Unadjusted Odds ratio	Adjusted Odds ratio	Adjusted *p*-value (Adjusted for study site)
Primary outcome: Composite outcome at day 90	186/339 (54.9)	184/348 (52.9)	0.92 (0.68, 1.25)	0.92 (0.68, 1.24)	0.572
Mortality by day 90	12/366 (3.3)	11/370 (3.0)	0.90 (0.39, 2.08)	0.90 (0.39, 2.08)	
Any hospital readmission before day 90	27/343 (7.9)	29/350 (8.3)	1.06 (0.61, 1.83)	1.05 (0.60, 1.82)	
Deterioration in nutritional status by day 90	164/327 (50.2)	154/336 (45.8)	0.84 (0.62, 1.14)	0.83 (0.61, 1.13)	
Composite outcome at day 180	224/312 (71.8)	225/334 (67.4)	0.81 (0.58, 1.14)	0.80 (0.57, 1.13)	
Mortality by day 180	16/363 (4.4)	12/368 (3.3)	0.73 (0.34, 1.57)	0.73 (0.34, 1.57)	
Any hospital readmission before 180 days	40/335 (11.9)	46/346 (13.3)	1.13 (0.72, 1.78)	1.15 (0.72, 1.83)	
Deterioration by day 180	187/294 (63.6)	189/318 (59.4)	0.84 (0.60, 1.16)	0.83 (0.59, 1.15)	

The median age of the participants who experienced adverse outcomes was 13 months (IQR 8, 21), and the majority were male, 253 (56.3%). The median weight-for-age baseline z score for these children was −0 (IQR − 1, 0), height-for-age baseline z score −1 (IQR − 1, 0), weight-for-length/height z-score −0 (IQR − 1, 1) and MUAC 15 cm (IQR 14, 16). There were relatively more children with grunting at baseline who experienced adverse outcomes 101/449 (22.5%) versus 29/197 (14.7%). However, there was generally an equal distribution of characteristics between participants who experienced adverse outcomes and those who did not (Table 3).

**Table 3:** Baseline characteristics of participants who experienced the composite adverse outcome in this study, Uganda and Kenya 2018–2022.

Parameter; *n* (%) or Median (Interquartile range) as stated	No (*N* = 197)	Yes (*N* = 449)	Adjusted odds ratio (CI)	*p*-value
Median age (months)	25 (15, 34)	13 (8, 21)	0.52 (0.45–0.62)	<0.001
Male Sex	118 (59.9)	253 (56.3)	1.31 (0.91–1.88)	0.147
Median Weight-for-age baseline z score	−1 (−2, −0)	−0 (−1, 0)		
Median Height-for-age baseline z score	−1 (−2, −0)	−1 (−1, 0)		
Median Weight-for-length/height z-score	−0 (−1, 0)	−0 (−1, 1)		
Median MUAC (cm)	15 (14, 16)	15 (14, 16)		
MUAC (categorized) ≥ 12.5 cm	186 (94.4)	435 (96.9)		
Fever (temperature >37.5 C)	91 (46.2)	204 (45.4)		
Median Initial SpO2 (first measurement)	89 (87, 90)	89 (87, 90)		
Median Respiratory rate (breaths per minute)	55 (48, 62)	56 (49, 63)		
Age-adjusted tachypnea	186 (94.4)	397 (88.4)		
Age-adjusted bradycardia	2 (1.0)	1 (0.2)		
Age-adjusted severe tachycardia	58 (29.4)	89 (19.9)		
Compensated shock (signs of impaired perfusion)	90 (45.7)	180 (40.1)		
Responsiveness: Alert	186 (94.4)	428 (95.3)		
Signs of current febrile illness (temperature>37.5 or <36)	93 (47.2)	215 (47.9)		
History of fever	187 (94.9)	404 (90.0)		
Difficulty breathing	194 (98.5)	439 (97.8)		
Vomiting or diarrhea	63 (32.0)	147 (33.0)		
Oral antibiotics or injections of antibiotics	102 (53.7)	249 (56.2)		
Used Inhalers or oral steroids, all children	12 (6.1)	20 (4.5)		
Grunting	29 (14.7)	101 (22.5)		
Crackles/crepitations on auscultation	145 (73.6)	348 (77.5)		
Audible wheeze on auscultation	55 (27.9)	132 (29.5)		
Gestation at birth ≥ 37 weeks	184 (94.8)	422 (94.6)		
Severe anemia	12 (6.1)	19 (4.3)		
Median WBC (10×3/μL)	12 (8, 15)	12 (9, 18)		
Leukocytosis	105 (53.6)	257 (58.0)		
Lactate ≥ 5 mmol/L	3 (1.6)	12 (2.8)		
Positive malaria rapid diagnostic test	58 (29.6)	95 (21.4)		
Positive malaria blood film	19 (9.7)	37 (8.3)		
Positive HIV at admission	2 (1.0)	8 (1.8)		
Sickle cell disease	17 (8.8)	41 (9.6)		
Radiology evidence of pneumonia	112 (56.9)	262 (59.5)		
Breastfeeding	180 (91.4)	411 (91.5)		
Immunization up to date	137 (90.7)	305 (90.2)		
Moderate malnutrition	11 (5.6)	14 (3.1)		
Median paediatric emergency triage (PET) score	2 (1, 3)	2 (1, 2)	1.11 (0.92–1.33)	0.282

The risk of experiencing adverse outcomes reduced with increasing age (adjusted odds ratio 0.53, 95% CI 0.45, 0.62, *p* < 0.001). Sex and PET score were not significantly associated with experiencing an adverse outcome, (adjusted odds ratio 1.31, (95% CI 0.91, 1.88), *p* = 0.147 and 1.11, (95% CI 0.92, 1.33), *p* = 0.282 respectively) (Table 3).

## Discussion

In this secondary analysis of the COAST-Nutrition trial, we specifically investigated the ability of 1 sachet of RUTF given daily for 8 weeks in addition to the usual diet to reduce adverse outcomes including mortality, readmission, and deterioration in nutritional status by day 90 compared to usual diet alone among undernourished children aged 6–59 months admitted for severe pneumonia. We found that a majority (54%) of undernourished children experienced adverse outcomes by day 90 of follow up and up to 70% experienced adverse outcomes by day 180. Supplementing the usual diet with RUTF did not reduce adverse outcomes in this population. The risk of experiencing adverse outcomes reduced with increasing age.

While the impact of RUTF on growth in children recovering from non-severe acute infections has been investigated with variable results (30, 31), there is a paucity of data among children with severe pneumonia. Macronutrient supplementation with modified foods as well as supplementary foods like RUTF have been explored among children with Moderate Acute Malnutrition (MAM), defined as weight-for-height/length > − 3 but ≤ − 2 in LMICs, leading to improved recovery rate from MAM and improved nutritional status (27, 29). Lipid-based nutritional supplements including RUTF did not significantly reduce mortality or progression to severe acute malnutrition among children with MAM (27). However, when compared to counseling alone, approximately 550 kcal/day of supplementary food (such as RUTF) for ≤ 12 weeks among children with MAM in Sierra Leone was protective from severe acute malnutrition and death (28).

Van der Kam et al. (30) showed that RUTF did not reduce the incidence of severe malnutrition during recovery among acutely ill Nigerian children without severe malnutrition, including those with pneumonia. This was thought to be possibly due to the short duration of intervention of 14 days (30). Nevertheless, our study gave the intervention (RUTF) for a longer period (8 weeks), close to that successfully used for MAM in Sierra Leone (28), but report similar results to the Nigerian study. Although the COAST Nutrition patients in this analysis were severely ill and undernourished (27, 3.6% with MAM) RUTF did not reduce adverse outcomes. This suggests that children with more severe forms of undernutrition (defined as a weight-for-age z score, height-for-age z score, or weight-for-height/length z score below −2 compared to the WHO reference population or mid-upper arm circumference (MUAC) below 12.5 cm) might benefit more from supplementation with RUTF as demonstrated by Rajabi et al. (28). It is possible that the nutritional requirements of children with severe pneumonia are not met by RUTF, since this food was specifically designed for severe malnutrition. Alternatively, their risk of adverse outcomes cannot be reversed solely by nutritional supplementation.

About 48% of participants in our study had a deterioration in nutritional status by day 90 of follow-up. This highlights the role of pneumonia as a causative or aggravating agent for undernutrition in children under 5 years. The fact that nutritional supplementation with RUTF did not reduce the risk of adverse outcomes among children with severe pneumonia underscores the need to strengthen primary prevention of undernutrition through exclusive breastfeeding, complementary feeding, and healthy young child feeding to prevent severe infections and adverse outcomes in children. Our finding that adverse events reduced with increasing age is in keeping with findings elsewhere (41, 42), and is probably due to the relatively less experienced immune system of younger children which increases their risk of severe disease.

To our knowledge, this is the first study to assess the effect of RUTF on undernourished children with severe pneumonia (complicated by hypoxemia) in LMICs. However, we acknowledge several limitations. The sample size was estimated to detect a large difference (58%) between the intervention and control arm. The study was therefore not powered to detect clinically significant differences smaller than 58%. Given the nature of the intervention, participants and health workers in the study were not blinded and this may have led to information bias. The participants included in this study were a subgroup of the larger randomized controlled trial population based on specific criteria, which may have introduced selection bias. However, there was no significant difference in baseline characteristics between intervention and control groups. Children with severe malnutrition were excluded from the main trial and this sub-analysis as it would have been unethical to randomize them to nutritional supplementation rather than therapeutic doses of RUTF. Adherence to RUTF, as well as food given as part of the usual diet, were not systematically assessed, so co-intervention may have occurred in the study thus diminishing differences between intervention and control groups. Finally, we were ethically required to give parents nutritional counseling in both arms (which is not routinely done in standard practice). This might have improved the nutritional practices in both arms and dampened the effect of the intervention.

## Conclusion

Our study showed that supplementation of the usual diet with ready-to-use therapeutic food (RUTF) for 8 weeks among undernourished children aged 6–59 months admitted for severe pneumonia did not reduce adverse outcomes (mortality, re-admission, and deterioration in nutritional status). Further studies using targeted nutritional supplementation specifically meeting the metabolic demands of under-nourished children aged 6–59 months recovering from severe pneumonia are recommended.

## Data Availability

The raw data supporting the conclusions of this article will be made available by the authors, without undue reservation.
